# Physical disturbance shapes vascular plant diversity more profoundly than fire in the sagebrush steppe of southeastern Idaho, U.S.A

**DOI:** 10.1002/ece3.574

**Published:** 2013-04-28

**Authors:** Matt Lavin, Tyler J Brummer, Ryan Quire, Bruce D Maxwell, Lisa J Rew

**Affiliations:** 1 Plant Sciences and Plant Pathology, Montana State UniversityBozeman, MT, 59717; 2 Land Resources and Environmental Sciences, Montana State UniversityBozeman, MT, 59717

**Keywords:** Amaranthaceae, *Artemisia arbuscula*, *Artemisia nova*, *Artemisia tridentata* spp. *wyomingensis*, Asteraceae, Chenopodiaceae, phylogenetic community ecology, phylogenetic niche conservatism

## Abstract

Fire is thought to profoundly change the ecology of the sagebrush steppe. The Idaho National Laboratory provides an ideal setting to compare the effects of fire and physical disturbance on plant diversity in high-native-cover sagebrush steppe. Seventy-eight 1-hectare transects were established along paved, green-striped, gravel, and two-track roads, in overgrazed rangeland, and within sagebrush steppe involving different fire histories. Transects were sampled for the diversity and abundance of all vascular plants. Alpha, beta, and phylogenetic beta diversity were analyzed as a response to fire and physical disturbance. Postfire vegetation readily rebounds to prefire levels of alpha plant diversity. Physical disturbance, in contrast, strongly shapes patterns of alpha, beta, and especially phylogenetic beta diversity much more profoundly than fire disturbance. If fire is a concern in the sagebrush steppe then the degree of physical-disturbance should be more so. This finding is probably not specific to the study area but applicable to the northern and eastern portions of the sagebrush biome, which is characterized by a pulse of spring moisture and cold mean minimum winter temperatures. The distinction of sagebrush steppe from Great Basin sagebrush should be revised especially with regard to reseeding efforts and the control of annual grasses.

## Introduction

Sagebrush vegetation embodies the wide-open spaces of western North America, yet it is better known for its state of decline (e.g., Carson 1962; Welch 2005; Davies et al. 2011). The combined effects of fire and physical disturbances have resulted in habitat fragmentation and loss that can only very slowly be reversed by natural seed immigration (e.g., Knick and Rotenberry 1997; Wambolt 2005). Pinyon–juniper (*Pinus monophylla* and *Juniperus osteosperma*) encroachment is blamed for losses of sagebrush vegetation in the Great Basin (e.g., Suring et al. 2005), but cheatgrass invasion via fire is considered generally most threatening (e.g., Suring et al. 2005; Mack 2011). Repeated fires may cause a change in sagebrush vegetation and eventually a change in state where the degraded state comprises a number of grass-dominated or invasive-dominated plant communities from which restoration to sagebrush vegetation is difficult (e.g., Jones and Monaco 2009; Davies et al. 2012).

Although fire disturbance receives much attention (e.g., Weltz et al. 2011), physical disturbance (e.g., removal of sagebrush and other shrubs) without fire may increase the growth rate and abundance of exotic forbs at the expense of natives, regardless of experimental effects on soil moisture (e.g., Prevéy et al. 2010a, b). Most notably, postfire reseeding efforts that cause physical disturbance can be counterproductive to the reestablishment of plant cover, density, and diversity in the sagebrush steppe (Ratzlaff and Anderson 1995).

We use the term “sagebrush steppe” as distinct from “Great Basin sagebrush” (sensu West and Young 2000) because the important conclusions of Ratzlaff and Anderson (1995) may apply to “sagebrush steppe,” which occurs at the most northern latitudes of the sagebrush biome where rhizomatous- and bunch-grass cover is high. Ratzlaff and Anderson (1995) and our observations of the sagebrush steppe suggest that fire and physical disturbance, each shape plant diversity in the sagebrush steppe in different ways.

**Figure 1:**
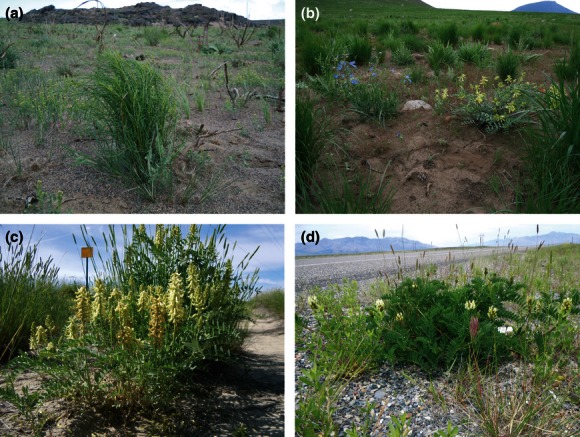
Representative *Astragalus* species common to the sagebrush steppe within the boundaries of the Idaho National Labs. 1.1. *Astragalus filipes* (behind the leaves of the native *Crepis acuminata*) growing in former high-native-cover sagebrush steppe during 2011 following a 2010 fire. 1.2. *Astragalus curvicarpus* (to the right of the blue-flowered native *Penstemon radicosus*) growing in former high-native-cover sagebrush steppe during 2011 following a 2007 fire. 1.3. *Astragalus canadensis* (in front of the exotic *Agropyron cristatum*) along a two-track road. *Astragalus cicer* (in front of the exotic *Phleum pretense*) growing along a paved road. *Astragalus filipes* and *A. curvicarpus* belong to the New World or Neo-*Astragalus* clade whereas *Astragalus canadensis* and *A. cicer* belong to Old World *Astragalus* groups (Barneby 1964; Wojciechowski et al. 1999). Photo-documentation of the plants in these INL settings is found at http://www.flickr.com/photos/plant_diversity/collections/72157631806074802/.

The Idaho National Laboratory (INL) harbors an accidental wilderness (Wolman 2010) including high-native-cover sagebrush steppe, which since World War II has not been exposed to cropping and only to limited grazing and construction. Fires, in contrast, have been common in recent decades but were infrequent during most of the 20th century. This study was motivated by our observations of vascular plant diversity in the sagebrush steppe both within and outside the INL. The patterns of occurrence of species in the genus *Astragalus* exemplify very well our hypothesis about the fundamental factors shaping plant biodiversity in the sagebrush steppe in southeastern Idaho. The genus *Astragalus* (Fabaceae) is species-rich in the North American sagebrush steppe (e.g., Barneby 1964, 1989; *Astragalus* nomenclature follows these sources). Certain species of *Astragalus* have diversified in semi-arid regions of North America over the last several million years and have base chromosome numbers of *x* = 10 and 11 (New World *Astragalus* or Neo-*Astragalus*; Barneby 1964; Wojciechowski et al. 1999), such as *Astragalus filipes* (Fig. 1a) and *A. curvicarpus* (Fig. 1b). Each New World *Astragalus* species has its closest relative within semi-arid vegetation of North American. Our observations suggest that the diversity and abundance of New World *Astragalus* is expected in the sagebrush steppe and that New World *Astragalus* species resprout or recolonize after fire in high-native-cover sagebrush steppe. In contrast, other *Astragalus* species have more recently arrived into North America and have a base chromosome number of *x* = 8, such as *Astragalus canadensis* (Fig. 1c) and *A. cicer* (Fig. 1d; introduced by Euro Americans), and have their closest relatives mostly in the Old World (e.g., *Astragalus canadensis* is arguably distinct from the widespread Asian *Astragalus uliginosus*). Our observations suggests that a diversity and abundance of Old World *Astragalus* species is expected not in high-native-cover sagebrush steppe but rather along roadsides and in overgrazed rangeland and pastures and that postfire conditions in high-native-cover sagebrush steppe do not appear to promote an abundance and diversity of Old World *Astragalus*.

Such observations of *Astragalus* and other higher-level vascular plant taxa warranted a study of how fire and physical disturbance each shape patterns of alpha, beta, and phylogenetic beta diversity. This requires a community phylogenetic approach (e.g., Webb et al. 2002, 2008) to test whether physical disturbance in the sagebrush steppe trumps fire disturbance in shaping patterns of species and higher-level plant taxonomic composition. The sagebrush steppe of the INL provided an ideal setting to test this hypothesis because of the vast tracts of physically undisturbed sagebrush steppe in this area that are bordered and divided by areas and roads that have experienced more frequent fire or physical disturbance. Our observations of *Astragalus* species suggest that the ability of a species to inhabit a certain position along a disturbance-stability gradient in a semi-arid environment is inherited, or phylogenetically niched conserved (e.g., Donoghue 2008). Distinct, evolutionarily determined assemblages of species can be expected at each end of the physical-disturbance gradient. Conversely, if a phylogenetic community signal is not detected, as we hypothesize with fire, then we can assume species in this environment readily adapt to such a disturbance. Thus, the outcomes of restoration efforts can be better predicted if we understand how profoundly fire and physical disturbances each structure the available species pools.

## Material and Methods

### Study site

**Figure 2:**
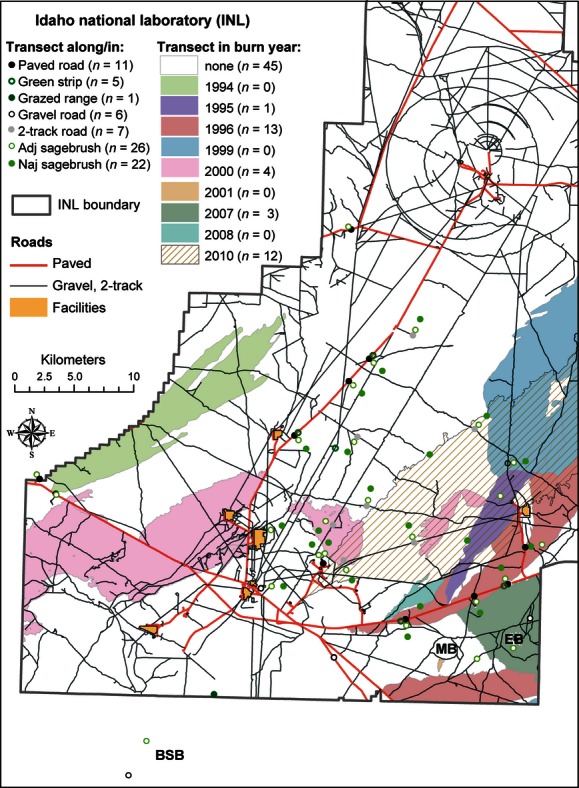
Transect locations in the INL and immediate vicinity. Three prominent buttes along the southern edge of the INL are Big Southern Butte (BSB), Middle Butte (MB), and East Butte (EB). The western-most set of two points sits at the very southeastern end of the Lost River Range. The northern-most set of two points sits at the very southern end of the Lemhi Range. Highway 20 is the main west-east paved road connecting Arco and Idaho Falls, Idaho. Lincoln Boulevard is the main paved road running northeast to southwest through the INL. Transects were located along paved road sides, green strips, in overgrazed rangeland (grazed range), along gravel and two-track roadsides, in sagebrush steppe adjacent to roads (Adj sagebrush), and in sagebrush steppe not adjacent to recently-used roads (Naj sagebrush). Samples sizes (n) are reported for each disturbance category.

A brief history of the INL lands is provided by Anderson and Inouye (2001). INL vegetation has been generally closed to grazing and most other large-scale human impacts since about World War II when it was used as a Naval gunnery range and then as a restricted research area. Although heavily cropped and grazed during the early 1900s, our reconnaissance and transect survey work during the summers of 2009, 2010, and 2011, during the peak flowering month of June, revealed large tracts of biodiverse high-native-cover sagebrush steppe. Reconnaissance work during 2009 and 2010 lead to the survey of 78 transects established mostly during 2011. These 78 spanned the elevation extent, 1459–1651 m, of open sagebrush steppe in the INL area. These transects were randomly positioned on the INL landscape following Brummer et al. (in press) and stratified among seven disturbance and six burn categories (Fig. 2). Transects were dominated by mostly *Artemisia tridentata* ssp. *wyomingensis* (Wyoming big sagebrush) with *Agropyron dasystachyum* codominant at the lower elevations and *Agropyron spicatum* codominant at the upper elevations. *Artemisia arbuscula* (little sagebrush), *A. nova* (black sagebrush), and *A. tripartita* (threetip sagebrush) each dominated or codominated in a few transects. Sites dominated by *Artemisia tridentata* ssp. *tridentata* (basin big sagebrush) and *Artemisia cana* (silver sagebrush) were not included because they involve a disturbance and soil-moisture regime that precluded the characteristic plant diversity found in the semi-arid short-statured sagebrush steppe (and such sites were uncommon in the INL area). Nomenclature follows mainly that of *The Intermountain Flora* (Cronquist et al. 1972; to Holmgren et al. 2012) and secondarily the USDA Plants Database (USDA, NRCS 2012).

A sampling transect of 1 km × 10 m was adopted from Rew et al. (2005) and Brummer et al. (in press) with three transect positions: adjacent to a road, perpendicular to a road but within 1 km or, >1 km from a road. To capture the local extent of plant biodiversity in a manner very similar to the Gentry transect (e.g., Phillips and Miller 2002) and the modified-Whittaker nested plots (Stohlgren et al. 1995; Seefeldt and McCoy 2003), we scored the occupancy of a species in twenty randomly located 10 × 10 m plots, such that a species occurring along a particular transect could have a maximum frequency of 20 and a minimum frequency of one.

Transects were geographically clustered such that adjacent transects differed in the dominant species of shrubby *Artemisia* or perennial grass, elevation, substrate, soil type, or disturbance or fire category (Fig. 2). In this manner pseudoreplication was minimized. All disturbance categories harbored shrubby *Artemisia* to the extent that they could be said to be currently or formerly sagebrush steppe but were also regularly (e.g., roadsides and rangeland) or once profoundly disturbed, as in the case of green strips (Pellant 1990) or burned areas. Transects adjacent to roadsides were selected only if they abutted and included high-native-cover sagebrush steppe. This biased our results away from detecting a difference between transects in sagebrush steppe and those along roadsides. In this context, appropriate transects located in overgrazed rangeland were difficult to find given that they were often lacking a sagebrush steppe physiognomy and included a paucity of Amaranthaceae shrubs (e.g., *Atriplex*, *Grayia*, and *Krascheninnikovia*; formerly Chenopodiaceae) and an abundance of annual exotic Amaranthaceae (e.g., *Halogeton* and *Kochia*). Thus, sampling of well-grazed rangeland, which was somewhat common along the very southern and western borders of the INL, was minimal. Sagebrush steppe sampled adjacent and insular to roads had ±100% native plant cover. This was considered indicative of infrequent disturbance during the past 50–100 years. Transect characteristics are posted to http://www.montana.edu/mlavin/data/INL_sagebrush_sites.txt.

Identification of plant species occurred in the field and at the Montana State University Herbarium (MONT) utilizing *The Intermountain Flora* as the main identification source. Herbarium specimens documenting the INL flora were studied at Gonzales-Stoller Surveillance, LLC (http://www.gsseser.com/CMP/Herbarium/herbariumsearch.asp), Idaho Falls, Idaho, and the Consortium of Pacific Northwest Herbaria (http://www.pnwherbaria.org/data/search.php).

### Alpha diversity

The effects of fire- and physical-disturbance on the alpha diversity of different plant functional groups and growth forms conventionally considered important (e.g., Anderson and Inouye 2001; Seefeldt and McCoy 2003; Davies et al. 2012; Prevéy et al. 2010a, b) were evaluated. Shannon's diversity index (H) was estimated for native species, introduced species, shrub species, graminoid species, herbaceous species, perennial species, annual species, species with tuberous roots (e.g., species of *Allium*, *Lomatium*, etc.) or other large or extensive underground storage organs (e.g., *Agropyron dasystachyum*, *Comandra umbellata*, etc.), species with hairy propagules (e.g., *Epilobium*, *Krascheninnikovia*, and Asteraceae bearing a capillary pappus), and species with air-dispersed propagules (e.g., all Orobanchaceae species; trait matrix: http://www.montana.edu/mlavin/data/sagebrush_sp_traits.txt). Although fire- and physical-disturbance have been implicated as having little effect on the alpha diversity of all vascular plants (e.g., Seefeldt and McCoy 2003), perhaps this is not necessarily the case for particular functional groups or growth forms.

### Explanatory variables

Fire disturbance included six categories and these were derived from a GIS layer indicating the year of the most recent burn (R. Blew and J. Shive, unpubl. data; Gonzales-Stoller Surveillance). Transects fell on sites with (1) no recent burn history (n = 45; sagebrush steppe dominated by *Artemisia tridentata*, *A. arbuscula*, or *A. nova*), (2) a 1995 burn (n = 1), (3) 1996 (n = 13), (4) 2000 (n = 4), (5) 2007 (n = 3), and (6) a 2010 fire (n = 12; Fig. 2). Only four transects were burned in more than one of these years so a parameter involving “multiple burns” was not used as an explanatory variable.

Physical disturbance included seven-categories. From frequently to infrequently disturbed, these included (1) along the sides of paved roads (n = 11 transects), (2) along green strips adjacent to paved roads (n = 5), (3) in overgrazed rangeland (n = 1), (4) along the sides of gravel roads (n = 6), (5) along the sides of two-track roads (n = 7), (6) in sagebrush steppe with high native plant cover immediately adjacent to roads or overgrazed rangeland (n = 26), and (7) in sagebrush steppe with high native plant cover not adjacent to roads or adjacent to roads with no evidence of having been used in recent history (n = 22; Fig. 2). In addition to physical- and fire-disturbance, other categorical variables recorded for each transect included the predominant soil texture (clay, silt, sand) and substrate (in the INL area: basalt, limestone).

Latitude and longitude (in decimal degrees) and elevation (m) were also attributed, as were bioclimatic variables (bio1–bio19), which showed sufficient variation within the study area and were thus evaluated as potential explanatory variables in competing models. These were obtained from the WorldClim 1.4 data layers (Hijmans et al. 2005) for each of the 78 transects using the ‘getData’ and ‘extract’ functions in the R package ‘raster’ (Hijmans and van Etten 2012). Of the 19 bioclimatic variables, 11 were measures of temperature (°C) and eight were measures of precipitation (enumerated in Lavin et al. 2013). All GIS data were WGS84 projected.

### Response variable

Mean-nearest-taxon (MNT) distances (Webb et al. 2002, 2008), a measure of phylogenetic beta diversity (Graham and Fine 2008), were the phylogenetic community distance modeled during this study. This metric involves the mean of the branch lengths that separate inter-site pairs of most closely related species (a species at one site is matched with its closest relative at another site until all species at two sites are paired). In contrast to phylosor, unifrac, and mean pairwise phylogenetic distances, the MNT metric is not scaled between 0 and 1 (as are phylosor and unifrac) and shows the greatest range of variation, much more than mean pairwise phylogenetic distances. MNT distances correspond well to Bray–Curtis distances derived from the differences in shared species and genera (http://www.montana.edu/mlavin/data/sagebrush_genus_19Sept2012.txt), whereas mean pairwise phylogenetic distance do not (Lavin et al. 2013). This is relevant given that differences in shared higher level taxa can serve as a phylogenetic proxy (e.g., Hamilton et al. 2012; Oliveira-Filho et al. 2013). Community phylogenetic distances were generated using the R statistical program version 2.15.2 (R development core team 2012) with the package ‘picante’ 1.4-1 (Kembel et al. 2012). The ‘comdistnt’ function in ‘picante’ uses the species occupancy matrix and the community phylogeny to produce a community phylogenetic distance matrix for all pairwise comparisons of sample sites. In addition to species abundances, MNT distances incorporated shared conspecifics between transects because we wanted to measure phylogenetic beta diversity at and above the species level with one composite metric.

The community phylogeny comprised 586 species sampled from 134 transects throughout Montana, Idaho, and Nevada (e.g., Lavin et al. 2013 and M. Lavin, unpubl. data) and was generated with Phylomatic (Webb and Donoghue 2005), Phylocom version 4.2 (Webb et al. 2008), and an angiosperm backbone tree (http://svn.phylodiversity.net/tot/megatrees/R20100701.new). The Phylomatic taxonomic list (http://www.montana.edu/mlavin/data/sb_phylomatic_19Sept2012.txt) followed the classification of Angiosperm Phylogeny Group (2009). Branch lengths were scaled to millions of years (Ma) using the branch-length-adjustment (bladj) option in Phylocom and the age estimates of Wikstrom et al. (2001) (http://svn.phylodiversity.net/tot/megatrees/ages). Our community phylogeny (http://www.montana.edu/mlavin/data/sagebrush_bladjtree_19Sept2012.txt) and species matrix (http://www.montana.edu/mlavin/data/sagebrush_species_19Sept2012.txt) were combined to generate community phylogenetic distances.

### Covariate selection

The explanatory power of categorical and continuous variables was evaluated using many approaches, which all yielded similar results (e.g., Lavin et al. 2013). We simplify the presentation by focusing on a multivariate approach. The 78 transects were arrayed by a principle coordinate analysis (PCO) of Euclidean (species frequencies) and MNT distances. The first two PCO axes, derived from both Euclidean and MNT distances, were used as response variables. The aforementioned categorical and continuous explanatory variables were initially evaluated separately. In each case, single parameter models were ranked in order to identify individual variables that were most explanatory of both species (Euclidean distances) and phylogenetic beta diversity (MNT distances) among transects. Continuous explanatory variables (e.g., latitude, longitude, elevation, and the 19 bioclimatic variables) were evaluated for collinearity using variance inflation factors (vif) implemented with the ‘corvif’ function in the R package AED (Zuur et al. 2009), whereby the variables with the highest factor was eliminated in a stepwise fashion until all remaining explanatory variables had a vif < 3. All remaining variables were then subjected to a stepwise model selection analysis using the function ‘dredge’ in the R package MuMIn (Bartoń 2013) and ‘bestglm’ function in the R package bestglm (McLeod and Xu 2012).

Models were ranked according to Akaike's information criterion for small sample sizes (AIC_c_, Akaike 1973) using the R packages ‘pgirmess’ version 1.5.4 (Giraudoux 2012), ‘bestglm’ and ‘MuMin’. Models with the lowest AIC_c_ value were considered the most explanatory. The relative plausibility of each model was evaluated by examining differences between the AIC_c_ value for the best model and values for every other model (ΔAIC_c_, Burnham and Anderson 2002; Johnson and Omland 2004). Models with ΔAIC_c_ < 2 were considered strongly supported by the data, and models with ΔAIC_c_ > 10 were considered to have essentially no support from the data. We measured the relative likelihood of a model given the data and the model list using Akaike weights (w_*i*c_), which are normalized values that sum to 1 across all models.

Variables identified as highly explanatory of patterns of beta diversity (Euclidean distances) had to remain consistently so of phylogenetic beta diversity (MNT distances). Such variables were then considered to represent ecologies that were evolutionarily important (e.g., a species had to have inherited its ability to cope with a particular spectrum of an ecological gradient rather than locally adapt to it). Such explanatory variables were plotted or contoured onto the two-dimensional PCO array using plotting and generalized additive modeling functions implemented in the R packages ‘vegan’ 2.0-4 (Oksanen et al. 2012) and ‘labdsv’ version 1.5-0 (Roberts 2012).

## Results

**Figure 3:**
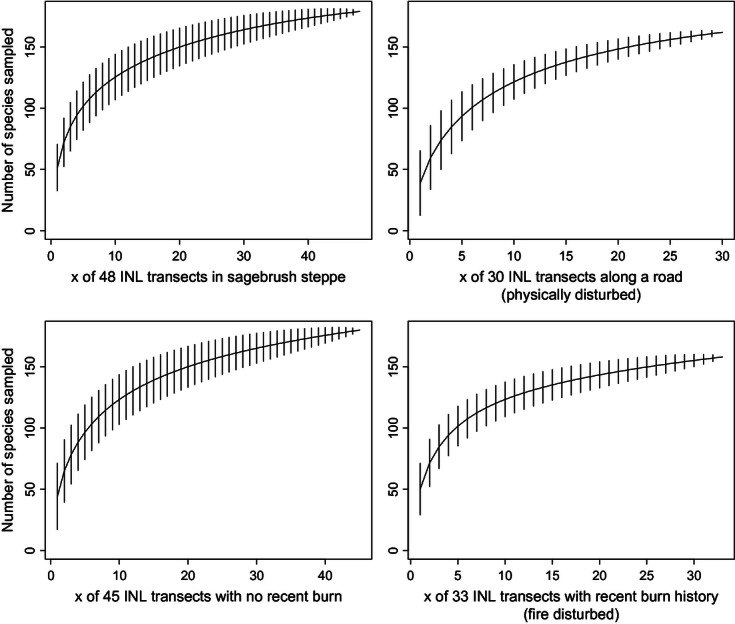
Species accumulation curves for subsets of the 78 INL transects. The upper two panels show subsets of physical disturbance (infrequently vs. frequently disturbed) and the lower two for subsets of fire disturbance (no vs. recent fire history).

From the seventy-eight 1-hectare transects, 203 vascular plant species were sampled (http://www.montana.edu/mlavin/data/INLrank_203species.txt), which represented 126 genera (http://www.montana.edu/mlavin/data/INLrank_126genera.txt). Species accumulation curves suggest that physical disturbance (i.e., roadsides versus not) more than fire disturbance (with or without evidence of recent fire) affects alpha diversity (Fig. 3; intercept) and that both kinds of disturbance may lower beta diversity (Fig. 3; slope). On average 39 (sd = 13) species were sampled per physically disturbed transect compared with 52 (sd = 10) species per infrequently disturbed transect, whereas 50 (sd = 11) species were sampled per transect with a recent fire history compared with 44 (sd = 14) in transects with no fire history.

### Alpha diversity

**Figure 4:**
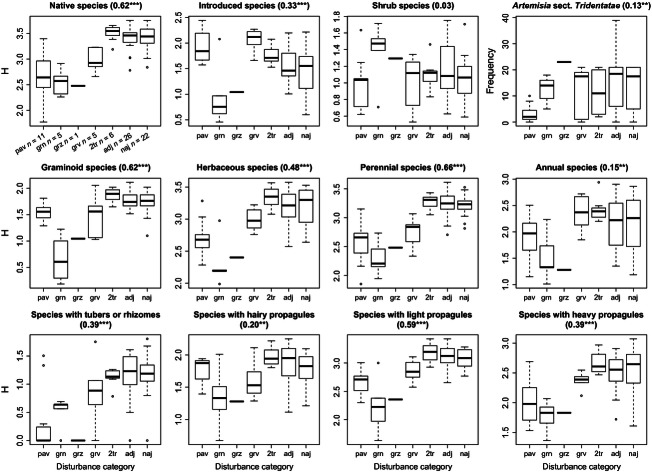
Ranges of Shannon's diversity index (H) of plant growth forms and the frequency of shrubby *Artemisia* (sect. *Tridentatae*) for transects classified among seven physical disturbance categories. These are arranged along the *x* axis from most to least physically disturbed: transects along paved roads (pav), transects along green strips (grn), transects in overgrazed rangeland (grz), transects along gravel roads (grv), transects along two-track roads (2tr), transects in sagebrush steppe adjacent to roads (adj), in sagebrush steppe not adjacent to roads (naj). The number of transects within each physical-disturbance category is listed in the upper-left-most panel. The number in parentheses in the title of each panel is the adjusted *R*^2^ for a model where physical-disturbance categories explain the alpha diversity of the featured functional group or growth form (the direction of influence is related to the trend in the boxplots). Significance of *R*^2^ is indicated as *P* > 0.05 (no asterisk or quote), *P* < 0.01 (*), *P* < 0.01 (**), and *P* < 0.001 (***).

The influence of physical disturbance on alpha diversity was pronounced with respect to native plant diversity (Fig. 4). Native plant diversity was generally higher in transects that were infrequently disturbed. Introduced species diversity had less of a trend with respect to physical disturbance categories. In general, either little trend in alpha diversity was detected for plant groups among disturbance categories or the sagebrush steppe was more diverse. Plant groups with alternative habits (e.g., herbaceous vs. graminoid, hairy vs. glabrous propagules, or light vs. heavy propagules) were each more diverse in the sagebrush steppe, for example (Fig. 4). The lack of strong trend between shrub species diversity or shrubby *Artemisia* (section *Tridentatae*) frequency and physical disturbance categories reflected our consistent sampling of a sagebrush steppe physiognomy regardless of disturbance category (Fig. 4).

**Figure 5:**
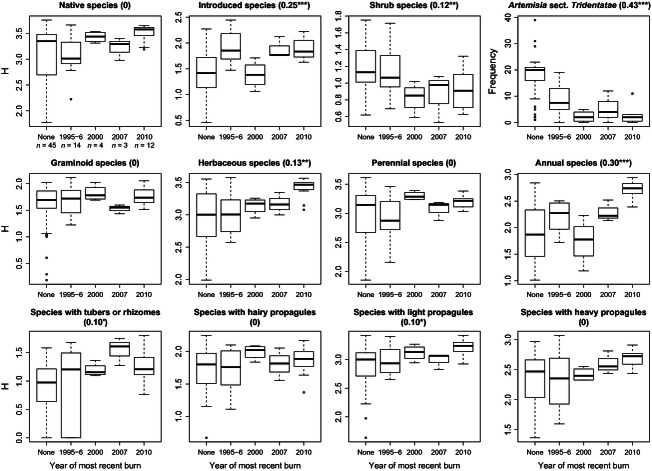
Ranges of Shannon's diversity index (H) of plant growth forms and the frequency of shrubby *Artemisia* (sect. *Tridentatae*) for transects classified by the year of the most recent fire. The number of transects within each fire-disturbance category is listed in the upper-left-most panel. The number in parentheses in the title of each panel is the adjusted *R*^2^ of a model where fire-disturbance categories explain the alpha diversity of the featured functional group or growth form (the direction of influence is related to the trend in the boxplots). Significance of *R*^2^ is indicated as *P* > 0.05 (no asterisk or quote), *P* = 0.1–0.05 (‘), *P* < 0.01 (*), *P* < 0.01 (**), and *P* < 0.001 (***).

Fire disturbance marginally if at all increased alpha diversity of most plant groups (Fig. 5; compare overall *R*^2^ values in Figs. 4, 5). Only shrub diversity may have been diminished by fire, which involved the reduction of the frequency of shrubby *Artemisia*. Recency of fire did not strongly increase the diversity of plants bearing tuberous roots (e.g., species of *Allium*, *Lomatium*, etc.) or well developed rhizomes (e.g., *Agropyron dasystachyum*, *Comandra umbellata*, etc.), especially when compared with the effects of physical disturbance (Figs. 4, 5). Alternative growth forms and habits such as native versus introduced, annual versus perennial, graminoid versus herbaceous, hairy versus glabrous propagules, and heavy versus light propagules showed no opposing trends with respect to fire disturbance (Fig. 5).

### Covariate selection

Analysis of variance inflation factors of the 19 bioclimatic variables along with elevation, latitude, and longitude of each transect resulted in the elimination of all but four of the continuous explanatory variables: longitude and bioclimatic variables 6, 9, and 18 (minimum temperature of coldest month, mean temperature of the driest quarter, and precipitation of the warmest quarter, respectively). Subjecting these four to a model selection analysis resulted in only bioclimatic parameter 18 being explanatory of PCO axis 1 for both Euclidean and MNT distances and bioclimatic parameter 6 for PCO axis 2 of both Euclidean and MNT distances. Geographic distance (latitude and longitude) between transects was expected to be little if at all explanatory given our sampling design of clustered transects that each differed in at least one significant ecological attribute.

Physical disturbance gradient was most explanatory of PCO axis 1 for both Euclidean and MNT distances (Tables 1, 2). Fire disturbance was most explanatory of PCO axis 2 for only Euclidean distances and was much less explanatory of this axis for with respect to MNT distances (Tables 3, 4). Although soil type was consistently but moderately explanatory of PCO axis 2 for both Euclidean and MNT distances, the main point here is that fire disturbance did not best explain beta and phylogenetic beta diversity along PCO axis 2.

**Table 1:** Rankings of covariates intended to explain beta diversity, the response variable principle coordinate analysis 1 (PCO1) of Euclidean distances (explaining 19% of the variation)

Covariate	*K*	ΔAIC_c_	Wi_c_	Adjusted *R*^2^
7 disturbance categories	8	0	1	0.61***
5 fire categories	6	51	0	0.23***
Bio18 – precipitation warm quarter	3	55	0	0.14***
3 soil categories	4	57	0	0.13**
2 substrate categories	3	66	0	0.02

The number of parameters (K), delta AIC_c_ for small sample sizes (ΔAIC_c_), and model weights for small sample sizes (Wi_c_) are reported.

Significance of *R*^2^ is indicated as *P* > 0.05 (no asterisk), *P* < 0.01 (**), and *P* < 0.001 (***).

**Table 2:** Rankings of covariates intended to explain phylogenetic beta diversity, the response variable principle coordinate analysis 1 (PCO1) of mean-nearest-taxon (MNT) distances (explaining 24% of the variation)

Covariate	*K*	ΔAIC_c_	Wi_c_	Adjusted *R*^2^
7 disturbance categories	8	0	1	0.65***
5 fire categories	6	68	0	0.13**
3 soil categories	4	68	0	0.09*
Bio18 – precipitation warm quarter	3	69	0	0.07*
2 substrate categories	3	69	0	0.06*

The number of parameters (K), delta AIC_c_ for small sample sizes (ΔAIC_c_), and model weights for small sample sizes (Wi_c_) are reported.

Significance of *R*^2^ is indicated as *P* > 0.01 (*), *P* < 0.01 (**), and *P* < 0.001 (***).

**Table 3:** Rankings of covariates intended to explain beta diversity, the response variable principle coordinate analysis 2 (PCO2) of Euclidean distances (explaining 15% of the variation)

Covariate	*K*	ΔAIC_c_	Wi_c_	Adjusted *R*^2^
5 fire categories	6	0	1	0.44***
3 soil categories	4	30	0	0.16***
7 disturbance categories	8	35	0	0.16**
Bio6 – minimum temp coldest month	3	36	0	0.07*
2 substrate categories	3	43	0	0

The number of parameters (*K*), delta AIC_c_ for small sample sizes (ΔAIC_c_), and model weights for small sample sizes (Wi_c_) are reported.

Significance of *R*^2^ is indicated as *P* > 0.05 (no asterisk), *P* < 0.01 (*), *P* < 0.01 (**), and *P* < 0.001 (***).

**Table 4:** Rankings of covariates intended to explain phylogenetic beta diversity, the response variable principle coordinate analysis 2 (PCO2) of mean-nearest-taxon (MNT) distances (explaining 19% of the variation)

Covariate	*K*	ΔAIC_c_	Wi_c_	Adjusted *R*^2^
3 soil categories	4	0	0.70	0.21***
Bio6 – minimum temp coldest month	3	2	0.25	0.17***
5 fire categories	6	5	0.5	0.18**
2 substrate categories	3	17	0	0
7 disturbance categories	8	26	0	0

The number of parameters (*K*), delta AIC_c_ for small sample sizes (ΔAIC_c_), and model weights for small sample sizes (Wi_c_) are reported.

Significance of *R*^2^ is indicated as *P* > 0.05 (no asterisk), *P* < 0.01 (**), and *P* < 0.001 (***).

**Figure 6:**
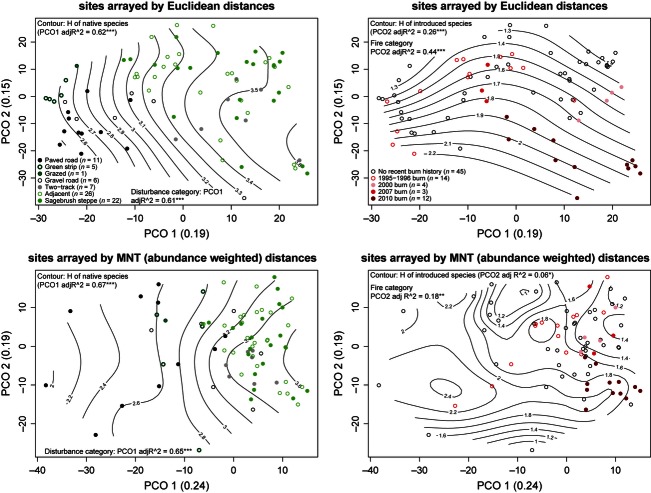
Principle coordinate (PCO) analysis of the 78 Idaho National Laboratory (INL) transects, which are ordinated along the first two axes using Euclidean distances (upper two panels) or Mean-nearest-taxon (MNT) distances (lower two panels). The proportion of variation captured by each principle coordinate analysis (PCO) axis is reported. Shannon's diversity of native species is contoured onto this surface in the two left-hand panels where the 78 transects are color-coded for disturbance category. Shannon's diversity of introduced species is contoured onto this surface in the two right-hand panels where the 78 transects are color-coded for burn history.

The consistent and highly explanatory nature of physical disturbance along PCO axis 1 for both Euclidean and MNT distances is graphically illustrated with PCO plots (Fig. 6; compare left two panels). In contrast, burn history was fairly explanatory of Euclidean distances among the 78 transects but not as much of MNT distances (Fig. 6; compare right two panels). The strong influence of physical disturbance on native plant diversity (Fig. 4) is graphically illustrated by the gam-fit contours of Shannon's diversity index of native species, which correspond strongly to PCO axis 1 for both Euclidean and MNT distances (Fig. 6; compare left two panels). In contrast, the moderate influence of fire disturbance on introduced plants (Fig. 5) is graphically illustrated by the gam-fit contours of Shannon's diversity index of introduced species, which more moderately corresponds to PCO axis 2 for Euclidean distances but less so to PCO axis 2 for MNT distances (Fig. 6; compare right two panels).

## Discussion

Our results suggest that within the sagebrush steppe of the INL area, alpha, beta, and phylogenetic beta diversity among sites are much more strongly related to physical disturbance than to fire. That phylogenetically distinct groups of plants occupied different ends of the disturbance-stability gradient (Fig. 6; lower left panel) suggests that a plant's ability to tolerate a particular physical-disturbance regime is more likely inherited than rapidly adapted to, which is evidence of phylogenetic niche conservatism (e.g., Donoghue 2008). Our results also suggest that observations of higher level taxa such as Old versus New World *Astragalus* apply generally to the sagebrush flora of the INL regions and perhaps elsewhere. Higher-level taxa generally have a predilection to a particular disturbance regime (Fig. 6, lower left panel). Consequently, selection of species for restoration purposes should focus on lineages preadapted to specific disturbance regimes, in addition to gene pools (e.g., Jones and Monaco 2009) and competitive genotypes (Goergen et al. 2011).

Fire disturbance was not detected as strongly influencing patterns of alpha, beta, and phylogenetic beta diversity (compare Figs. 5; Fig. 6, right two panels), which suggested that fire represents a disturbance regime to which many sagebrush plants can easily adapt, a finding also found for plant lineages inhabiting the fire-prone South American cerrado (Simon and Pennington 2012; Hughes et al. 2013). Our results agree with Ratzlaff and Anderson (1995), who suggested that postfire recovery of sagebrush steppe to a high diversity of native herb, grass, and shrub species does not require augmentation by reseeding programs because the physical disturbance involved may impede natural recovery. It may well be the case that alpha, beta, and phylogenetic beta diversity of postfire plant communities in the sagebrush steppe is more dependent on the prefire physical-disturbance regime than on fire itself (Fig. 6; compare two lower panels), a finding that agrees with that of Seefeldt and McCoy (2003).

### Plant biodiversity in the sagebrush steppe

This study represents one of the few with a focus on plant diversity in the sagebrush steppe. Welch (2005) overviewed the impacts of sagebrush steppe fragmentation on the biodiversity of mainly animals. Anderson and Inouye (2001) analyzed long-term plant diversity data from the INL sagebrush and found an increase in both alpha and beta diversity over a 45 year period, which suggested that as sagebrush steppe recovers from physical disturbance (i.e., early 1900s cropping and grazing), it tends toward a dynamic biodiversity equilibrium and not a static climax community. They found that the plant diversity of the kind harbored by the INL sagebrush steppe was largely resistant to colonization of introduced species and dominance of *Bromus tectorum* (cheatgrass). Our results are in agreement, especially for sagebrush steppe away from the well-maintained roads and overgrazed rangelands.

We found alpha diversity of all species to be relatively unaffected by physical or fire disturbance (Fig. 3), which agrees with Seefeldt and McCoy (2003). Alpha diversity of native plant species, however, was most affected by physical disturbance (Fig. 4). Indeed, the general increase in alpha diversity detected for many plant functional groups and growth forms with respect to infrequent physical disturbance regimes (Fig. 4) is likely related to the increase in native plant diversity. This is suggested by plants groups with theoretically alternative traits, such as plant groups marked by airborne propagules versus gravity-dispersed propagules, which are both more diverse in settings with an infrequent physical disturbance regime.

**Figure 7:**
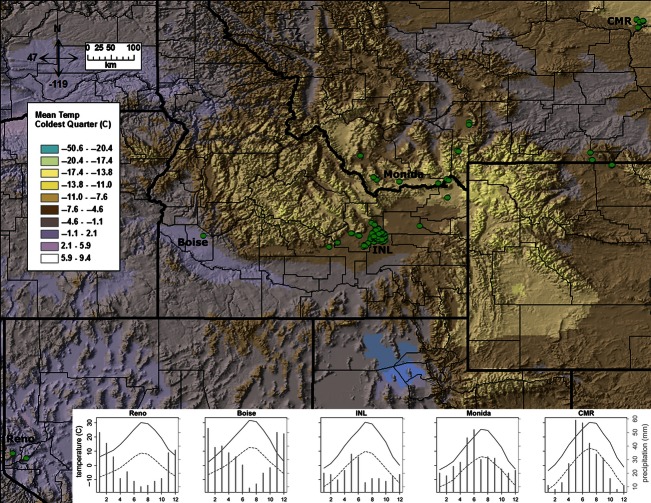
Map of sagebrush study sites (Lavin et al. 2013 and M. Lavin, unpubl. data) along a megatransect that bisects the sagebrush biome from the foothills of the Carson Range near Reno, Nevada, northeast to the Charles M. Russell National Wildlife Refuge, Phillips County, Montana. Climate diagrams are arranged from left to right: Reno (Thomas Creek, Nevada, 1672 m, −119.83020, 39.39801), Boise (Table Rock, Idaho, 983 m,−116.15380, 43.60524), Idaho National Laboratory (INL) (near Atomic City, Idaho, 1509 m, −112.85510, 43.47758), Monida (Monida Pass, Montana, 2071 m, −112.37410, 44.65242), CMR (Reynolds Road, Montana, 786 m, −107.67470, 47.75141). For the climate diagrams, bars = mean monthly precipitation (right-hand *y* axis), upper solid line = mean monthly temperature highs, and lower dashed line = mean monthly temperature lows (left-hand *y* axis). January through December are indicated numerically along the *x* axis. Bioclimatic variable 11, mean temperature during the coldest quarter, is contoured on the map. This contour and the climate diagrams suggest that the INL region is more climatically similar to sagebrush areas to the north and east than to the south and west.

In contrast to alpha diversity, patterns of beta and phylogenetic beta diversity (e.g., Fig. 7) are more informative about the profound effects of physical disturbance. The turnover of species and higher taxa is much greater among transects belonging to different physical-disturbance categories (Fig. 6; PCO axis 1 in both left-hand panels) than to different fire categories (Fig. 6; PCO axis 2 in both right-hand panels).

*The Intermountain Flora* (Cronquist et al. 1972; to Holmgren et al. 2012) centerpieces the high levels of plant diversity and endemism in an area of western North America that is dominated by short-statured sagebrush. In contrast, the sagebrush steppe has been reported to harbor only moderate levels of plant diversity (e.g., West and Young 2000). Perceptions of low to moderate plant diversity in the sagebrush steppe may be a product of most studies involving degraded sagebrush steppe or an analytical focus that ignores rare species (e.g., <1–5% cover; e.g., Davies et al. 2012; McCune and Grace 2002). Our results agree with *The Intermountain Flora* and underscore the high levels of plant diversity in high-native-cover sagebrush steppe (e.g., Fig. 3). Furthermore, such high levels of plant diversity can be fully utilized in a community phylogenetic analysis because the many rare or singleton species (often herbs) will have close relatives among the total sample of species, and thus will be informative of regional patterns of phylogenetic beta diversity. This is illustrated by our finding that more variation was captured along the first two PCO axes when MNT rather than Euclidean distances were used (Fig. 6).

### Ecological stability of the sagebrush steppe

Native diversity was higher in more stable sites and lower in more physically disturbed sites. This may be a reflection of the sagebrush steppe representing one of the most ecologically stable forms of northern latitude vegetation under historic disturbance regimes and, therefore, not prone to immigration by colonizing species. Climate data extracted from WorldClim (Hijmans et al. 2005) revealed that sagebrush steppe is either in water deficit or frozen for much of the year (Fig. 7; climate diagrams). Long-lived perennial plant species are common to this type of sagebrush steppe (e.g., Wambolt and Watts 1996; West and Young 2000; Wambolt and Hoffman 2001; Cooper et al. 2011) and this axiomatically suggests that they have evolved to survive in an environment where the favorable season is short, erratic, and unpredictable. Regular or occasionally profound physical disturbances would lower the probability of sagebrush-steppe-adapted plants amortizing the cost of growing new leaves and other photosynthetic organs when climatic conditions do not allow for much of a growing season. Such a climatically harsh environment that includes a high rate of physical disturbance would favor a completely different suite of species (e.g., a diversity of annual Amaranthaceae, Brassicaceae, and Poaceae) than what predominates in high-native-cover sagebrush steppe.

In contrast, introduced plant species diversity was not as well explained by the physical disturbance gradient, with introduced species occupying both roadside and high-native-cover sagebrush areas. We found introduced species to be abundant and diverse in physically disturbed settings, but they were also diverse, though were less abundant, in the stable sagebrush steppe. Within high-native-cover sagebrush steppe, small patches of regular physical disturbances (e.g., localized small-mammal burrowing, trampling, grazing, etc.) harbor a diversity of introduced species (e.g., annual Brassicaceae), as well as natives plants (e.g., annual species of *Cryptantha*, *Lappula*, and *Mentzelia*). Regularly disturbed roadsides may well be serving as conduits for invasion into small disturbed patches within the stable sagebrush matrix. However, stable patches do not similarly exist in regularly disturbed roadsides and rangeland, so native species characteristic of stable conditions do not often invade (or reinvade) these disturbed ecologies, especially those well-traveled and well-grazed. This explains why native species diversity tracks physical disturbance much more strongly than introduced species diversity in the sagebrush steppe.

These findings have distinct implications concerning the choice of species used in restoration projects. Physical and fire disturbances continue to push sagebrush steppe across thresholds from which restoration to a predisturbance state is becoming increasingly difficult (e.g., Davies et al. 2011) and has prompted the consideration of primary, secondary, tertiary, and quaternary gene pools of potential restoration species (e.g., Jones and Monaco 2009). Primary gene pools come from locally adapted populations of the candidate restoration species whereas the quaternary restoration gene pool comes from a surrogate species of the same functional group when the original species is no longer adapted to the modified (degraded) environment. If physical disturbance in short-statured sagebrush steppe imposes an evolutionary impediment to adaptation as our results suggest, then species pools also should be considered when choosing plant material for restoration. In order for the expectations of restoration outcomes to be realistic, practitioners must consider the evolutionary limitations of plant lineages that disturbance regimes impose, and either choose species that can inhabit those conditions, or drastically alter the disturbance regime to favor a level of stability that is associated with the desired species that often represent the restoration targets.

Plant taxa that represent reclamation and restoration targets of physically disturbed sagebrush steppe have been enumerated briefly Lavin et al. (2013). Suffice it to say that restoration benchmarks for sagebrush steppe should include a diversity of shrubs in the Amaranthaceae and Asteraceae, succulents in the Cactaceae and Crassulaceae, and Portulaceae, native bunchgrasses belonging to the tribes Stipeae (needlegrasses) and Triticeae (wheatgrasses), and herbaceous species including New World *Astragalus*, Orobanchaceae (especially *Castilleja*, *Cordylanthus*, and *Orthocarpus*), *Eriogonum* and *Oxytheca* (Polygonaceae), dry-adapted Apiaceae (e.g., *Cymopterus*, *Lomatium*, *Perideridia*, and *Pteryxia*), and perennial *Cryptantha* (Boraginaceae). Because of ecosystem stability requirements, these taxa may not be the best candidates for initial introduction into a degraded sagebrush steppe. More disturbance-tolerant taxa affiliated with open dry environments might serve this purpose better. Such taxa could include root-sprouting shrub species of the genera *Artemisia*, *Chrysothamnus*, *Ericameria*, and *Tetradymia* (e.g., Davies et al. 2011). Such potential candidates for reclamation would most likely respond to the immediate attentions of reclamation efforts.

The idea that communities with more species are more resistant to invasion is supported by our results. Phylogenetic beta diversity patterns suggest that high-native-cover sagebrush steppe is resistant to invasion by higher-level taxa such as Cleomaceae, Euphorbiaceae, introduced Amaranthaceae, and introduced and native species of the family Fabaceae and the genus *Bromus*. *Bromus*, according to our studies (e.g., Lavin et al. 2013), for example, is a higher-level taxon with a predilection for disturbance-prone settings. *Bromus inermis* and *B. japonicus*, for example, are found along the paved roads in the INL region but are rarely if ever found in the adjacent INL sagebrush steppe. Even *Bromus tectorum*, although occurring through much of the INL area, is often at low abundance within high-native-cover sagebrush steppe, a finding consistent with Anderson and Inouye (2001). We suggest that the mechanism of resistance to invasion is not that the sagebrush steppe is more diverse, as postulated by the diversity-invasibility hypothesis (e.g., Fridley 2011), but that it is more stable. The diversity-invasibility hypothesis suggests that new species are precluded because they cannot find resources to utilize because all the niches are filled. The results of our study suggest that colonizing species cannot successfully immigrate and replace the plant species adapted to the sagebrush steppe because they are not adapted to stable conditions and thus end up being precluded from the species pool. At higher taxonomic levels, most Fabaceae and all Cleomaceae and Euphorbiaceae, for example, will not thrive in the stable sagebrush steppe because such a setting does not cater to colonizing and disturbance-tolerant species that respond opportunistically to the immediate growing conditions.

### Green stripping

The introduction of crested wheatgrass (*Agropyron cristatum*) is considered as an impediment to the restoration of sagebrush steppe. It can potentially resist grazing (e.g., Ganskopp et al. 1992) and dominate the seed bank (e.g., Marlette and Anderson 1986) in vegetation that otherwise would be characteristic of sagebrush steppe. Where the probability of restoration success is low and the threat of annual grass invasion high in Wyoming sagebrush steppe, crested wheatgrass has been suggested as a practical restoration alternative (Asay et al. 2001). Davies et al. (2011) go so far as to suggest that ecological processes in sagebrush plant communities may not be substantively changed when native perennial bunchgrasses are replaced with crested wheatgrass. Our results suggest that a slightly more nuanced view is required.

Our sampling of the INL green strips suggests that where livestock grazing is rare if at all present (as in the INL), crested-wheatgrass-dominated vegetation may provide the stability necessary for a diversity of Asteraceae and Amaranthaceae shrubs to assemble. Although green-strip transects were generally low in plant diversity and thus low in many plant functional types, they were notably diverse in shrub species (Fig. 4; panels labeled “shrub species” and “*Artemisia* sect. *Tridentatae*”). Overall, the plant composition and diversity of green-strip transects was low and very different from those of the high-native-cover sagebrush steppe with respect to Euclidean distances (Fig. 6; upper left, dark green open circles). With MNT distances, green-strips harboring a diversity of shrubby Asteraceae and Amaranthaceae were not as different from the sagebrush steppe transects relative to some of the paved road transects (Fig. 6; lower left, dark green open circles). These results suggest that if crested-wheatgrass-dominated vegetation is subjected to little if any livestock grazing, a diversity of semi-arid-adapted shrubs including those palatable Amaranthaceae can successfully immigrate and establish in this ecological setting, whereas exotic annuals including *Bromus tectorum* cannot. Perhaps stands of *Agropyron cristatum*, in cases of highly degraded sagebrush steppe, can give the required levels of stability needed to restore Wyoming big sagebrush steppe. From a management perspective, the duration of that stability would have to encompass many decades if not over a century especially for Wyoming big sagebrush steppe (e.g., Anderson and Inouye 2001; Monsen 2004; Cooper et al. 2011; Davies et al. 2011; Kay and Reid 2011) and presumably for other short-statured forms of this vegetation.

## Conclusion

The observation that a physical disturbance gradient in the western North American sagebrush steppe shapes patterns of beta and phylogenetic beta diversity is not likely to translate to other biomes in temperate latitudes of North America. Riparian-wetland settings, for example, are prone to floods and drought, rendering disturbance-prone plant communities similar at higher taxonomic levels to those found along an adjacent road (e.g., Hood and Naiman 2000). The occasional mass mortality that is expected of plant communities in both riparian and road corridors favors the successful immigration and establishment of opportunistic colonizers. Road construction, high rates of livestock grazing, oil and gas development, and urbanization in the area of the sagebrush steppe of western North America have introduced unprecedented levels of physical disturbance to this biome, thus shifting the system to states that favor a wholly different assemblage of plant lineages. The effort to restore frost-tolerant and drought-adapted short-statured sagebrush steppe (e.g., Davies et al. 2011) will require long periods of stability to allow its adapted plant lineages to reestablish and dominate. Restoration planners in these systems must very carefully consider the physical disturbance regime before implementing management. Otherwise restoration attempts may be doomed due to a lack of site stability that inevitably favors nonsagebrush-steppe-adapted lineages of plant species.

Our finding that fire by itself represents a relatively weak force in changing the fundamental species pool adapted to the sagebrush steppe may not be unique to the INL area. The climate of this area involves low winter precipitation and very cold winter temperatures, which do not favor the winter growth of *Bromus tectorum*. Such INL climatic conditions are found more to the north and east than to the south and west in the sagebrush biome (Fig. 7). Climate conditions alone, even without the consideration of winter-active soil crusts (e.g., Kuskel et al. 2012), may explain why *Bromus tectorum* and potentially other winter annuals do not predominate in the sagebrush steppe to the north and east of the INL area as they do to the south and west including lower elevations of southwestern Idaho. The findings of Ratzlaff and Anderson (1995) concerning physical disturbance caused by reseeding efforts in postfire sagebrush steppe actually hindering the natural re-establishment of desirable native plant species are applicable to southeastern but not southwestern Idaho. The distinction of the sagebrush steppe from Great Basin sagebrush (West and Young 2000) should be revised, therefore, by distinguishing the sagebrush steppe in the north and eastern portions of the sagebrush biome by the inability of cheatgrass (and other species in *Bromus* section *Genea*) to invade it following physical and fire disturbance.
